# Sensing Home: A Cost-Effective Design for Smart Home via Heterogeneous Wireless Networks

**DOI:** 10.3390/s151229797

**Published:** 2015-12-03

**Authors:** Xiaohu Fan, Hao Huang, Shipeng Qi, Xincheng Luo, Jing Zeng, Qubo Xie, Changsheng Xie

**Affiliations:** 1 School of Computer Science and Technology, Huazhong University of Science and Technology, Wuhan 430074, China; qishipeng@hust.edu.cn (S.Q.); luoxc613@hust.edu.cn (X.L.); zengjing@hust.edu.cn (J.Z.); baidu@hust.edu.cn (Q.X.); 2 School of Software Engineering, Huazhong University of Science and Technology, Wuhan 430074, China; 3 Wuhan National Laboratory for Optoelectronics, No. 1037 Luoyu Road, Wuhan 430074, China

**Keywords:** activity recognition, pervasive computing, cost-effective, mobile integration

## Abstract

The aging population has inspired the marketing of advanced real time devices for home health care, more and more wearable devices and mobile applications, which have emerged in this field. However, to properly collect behavior information, accurately recognize human activities, and deploy the whole system in a real living environment is a challenging task. In this paper, we propose a feasible wireless-based solution to deploy a data collection scheme, activity recognition model, feedback control and mobile integration via heterogeneous networks. We compared and found a suitable algorithm that can be run on cost-efficient embedded devices. Specifically, we use the Super Set Transformation method to map the raw data into a sparse binary matrix. Furthermore, designed front-end devices of low power consumption gather the living data of the habitant via ZigBee to reduce the burden of wiring work. Finally, we evaluated our approach and show it can achieve a theoretical time-slice accuracy of 98%. The mapping solution we propose is compatible with more wearable devices and mobile apps.

## 1. Introduction

Activity recognition technology is critical to many human-centric ubiquitous applications [1]. It has been estimated that over one billion humans will be over 60 years of age by the year 2025 [2]. At the same time, the development of the mobile internet has facilitated health service and home care. Increasing requirements of more effective ways of providing care and service to the disabled and elderly at home, in addition to a smarter private pervasive environment in the home setting, will come closer to people’s daily lives. Google has invested in intelligent temperature controllers, with the acquisition of Nest at a price of $3.2 billion [3], which indicates that great opportunity and markets will emerge in the future. Wearable devices and mobile applications have already entered every aspect of human life. Smart spaces are complex computing systems that intimately fuse devices, humans as well as things, closely related to ubiquitous computing, communication and control technologies [4].

In response to these emerging opportunities, researchers have put forward a variety of approaches to model and recognize activities. Homes as the minimal living units of the real human world, play an important role in human behavior, because nearly half or more of the time and activities of the elders take place at home. Pervasive healthcare systems facilitate various aspects of research, including sensor technology, software and network architecture, machine learning algorithms, artificial intelligence and human-computer interaction. Activities of daily living (ADLs) [5], such as sleeping, bathing, cooking, and so on, are good indicators of cognitive and physical capabilities [6]. While computer vision-based machine recognition methods are difficult to resolve due to the nature that the activity poses, a computer vision-based solution requires higher storage and process cost, plus the wiring and location selection rely on professional construction personnel.

Simple binary sensors with advanced features of easy wiring, low cost, high robustness, low power consumption, strong expansion, high accuracy, and so on, seem to be enough to cover the home-level human activities detection [7]. As a solution, many research groups have shared their activity datasets [8,9,10,11,12,13,14], and many accurate offline learning models [12,13,15,16] have been put forward. However, so far there has been little work done on effective real-time classification and activity prediction solutions on embedded devices. In addition, most traditional smart home environments require complicated decoration and wiring work, so for putting up installations to deploy the sensors and controllers at the real home setting, the cost of re-decoration and human resources is huge, which hurts user satisfaction at the very beginning. Besides, the actual accuracy of activity recognition is low because the home environment settings and habits of each householder vary, so there is no general solution but to use actual activities’ living data as the training set for pattern recognition. In this paper, we will propose a practical method to deploy a perceived family environment based on ZigBee, Wi-Fi and sensor technologies such as a private smart space. In this way, the traditional wiring and decoration complexity were reduced and the system is easily installed and attached to the wall, and able to work with batteries. Humidity, gas, smoke, temperature, pressure, illumination and passive infra-red (PIR) sensors are applied for designing and developing our sensing home system in this paper. We tried to monitor the behaviors of the residents to analyse the abnormal events, such as heart attacks, hypertension or falls, to inform the appropriate people to check the situation and administer first aid if necessary. A system prototype is proposed and the experimental results are discussed, including a new type of ADL records, involving time value in the computation in order to enhance the accuracy, and choose a portable algorithm for behavior recognition and feedback control policy. Thus, a cost-effective ARM-based home server capable of working with lower power consumption is devised. A mobile app for Android is provided, which integrates initial configuration, electrical appliances control, an alarm message, environmental status and optional vision-based surveillance together, and it should prove compatible with many wearable devices in the future.

The main contribution of this paper is: (1) its focus on features for elderly people, comparing many famous smart home schemes and figuring out two different binary ADL datasets, and running various algorithms to find the key factors that affect the discriminant household behavior; (2) a proposal for reasonable deployment, and how to use the cost-effective equipment to realize the intelligent home furnishing sensor deployment and implementation details; (3) we put forward the new ALD records type, which reconstructs the original polynomial sensor and activity dataset into a larger sparse binary type coordinate with time line. In this way, data gathered from sensors will be easily recorded, and more importantly, computational complexity was reduced and coarse-grained methods enabled, at the expense of tolerable larger occupied memory; (4) for the experiments, we used open datasets for a theoretical accuracy test, and actually deployed our prototype to verify the scheme; (5) finally, we found a suitable way for real-time reaction by a private smart space with high accuracy in a home setting via a heterogeneous wireless network. The prototype can be applied in an embedded device based on TI-cc2530 and Raspberry Pi, which is compatible with wearable devices and smart appliances, integrated with a mobile app for remote surveillance and to recognize the behavior of the residents.

The remainder of the paper is organized as follows: in Section 2, we introduce the related work about the design and applications in a smart home. In Section 3, we describe the theoretical fundamental hypothesis of the behavior routine, and the selection of the sensor and the hardware devices. Subsequently in Section 4, the prototype, heterogeneous architecture process is described in detail. Section 5 gives a full implementation and results to verify our design with home health care applications and mobile applications. Finally, we summarize the discussions and give some conclusions in Section 6.

## 2. Related Works

Recognizing human activities from video is one of the most promising applications of computer vision, and reference [17] has elaborated on the forefront of the advances in the field. Robustness, real-time performance, high processing and storage costs, and intellectual challenges are the main difficulties.

In this paper, however, we focus on binary sensors which collect ADLs’ streaming sensor records and human activities. Reference [18] described the design and collection methods for ADLs, and we modified the scheme to construct our wireless solution. van Kasteren implemented the temporal probabilistic hidden Markov model (HMM) and conditional random fields (CRF) algorithms [12] to recognize activities from sensor readings, dividing time slices and labels for each. MIT provided three datasets called the PlaceLab Intensive Activity Dataset [8] with more fine-grained algorithms for accurate identification and achieved higher accuracy, but this kind of environment seems perfect yet difficult to deploy in a real home. In the CASAS project [11], the AI-Lab of Washington State University provides several activity datasets and made progress on partnership learning using this CASAS dataset [15] and a web based simulator named Persim for synthesis applications. The main smart homes and ADLs research projects and websites are listed as follows:

MIT House_n: Http://web.mit.edu/cron/group/house_n/ [8]
CASAS: http://ailab.wsu.edu/casas/datasets.html [11]
Smart Umass: http://traces.cs.umass.edu/index.php/Smart/Smart [15]
Adaptive House, University of Colorado Http://www.cs.cp;pradp.edu/~mozer/nnh/ [19]
Carnegie Mellon’s Intelligent Workspace Http://www.arc.cmu.edu/cbpd/iw/ [20]
Duke University Smart House Http://smarthome.duke.edu/ [21]
Georgia Tech Aware Home http://awarehome.imtc.gatech.edu/ [22]
MavHome at University of Texas Arlington Http://ailab.wus.edu/mavhome/ [23]
Smart Medical Home Http://www.urmc.rochester.edu/ [24]
GETALP http://getalp.imag.fr/xwiki/bin/view/HISData/ [25]
UCI Machine Learning Repo http://archive.ics.uci.edu/ml/datasets.html [26]

Several research projects have proposed a home energy management system (HEMS) [27], or cost-effective ecosystem to reduce power consumption [28], and improve the demand response system to accommodate user preference changes and satisfaction. Chen [29] proposed a remarkable Digital Signal Processor and Wi-Fi framework to monitor environment information to refer to, without human behavior detection. In China, taking the average economic level and the amount of population into account, there are 0.2 billion people over 60 this year with a gene coefficient of 4.7. Besides, the design structure of real estate is different from thst used in Western countries, and the popularity of renewable energy generating equipment is insufficient to deploy theoretical cost-effective models.

Research on a systematic methodology for smart spaces has been provided [30], and the quality of experience refers to the factor of human impact on design and perception, experience and expectations of the whole performance [31,32]. Despite the various smart home applications that have been developed, most of them are application specific and lack a systematic design method. Fortunately, while mobile Internet applications, IPTV, and wearable smart devices are varied and isolated, but most of them adopt a standard communication protocol like Bluetooth, ZigBee or Wi-Fi. Therefore, an effective, reliable and user-friendly design related to ubiquitous computing, control technologies and a knowledge-driven design would be a suitable but challenging task. Compatibility with numerous sensors and appliances is complex. Thus, two mapping procedures of both sensors and electric appliances are required for our system at the configuration phrase.

More recent research has tapped into the use of mobile phones or wearable sensors with accelerometer and gyroscope capabilities [33,34], indoor location tracking [35] and a heterogeneous network application [36]. These new sampling methods of sensors extend the data dimensions with extra features of the resident, which enable a more complex analysis of human behavior for our future work.

## 3. Preliminary

Before we start, some fundamental preparation work is necessary, including sensor selection, a deployment scheme, feature extraction, algorithm selection and validation of effectiveness. Referring to the solution mentioned in Section 2, we chose proper sensors capable of smart home environment monitoring and activity surveillance to deploy our wireless prototypes. Taking cost-effectiveness into account, we use an ARM-based embedded server to replace the PC-based workstation for its lower power consumption. Then, in order to implement remote control easily, our prototype should be able to seamlessly connect with a mobile application.

### 3.1. Comparison for Deployment

For deployment in a real home setting environment, we compared many well-known projects, such as the MIT house_n, CASAS, Kasteren and Ordonez, to assess their sensor layout and the accuracy of classifiers. A comparison of different schemes mentioned above is shown in Table 1, below. The MIT and CASAS prototypes use too many sensors, and indeed achieved higher accuracy, but they are inconvenient to deploy. In addition, most sensors in the Kasteren dataset are related with a door trigger, so there might naturally be more noise, and might lead to a probabilistic model application only, insufficient for monitoring human activities in real-time.

**Table 1:** Scheme Comparison.

Datasets	MIT	CASAS	Ordonez	Kasteren
Sensors	77 to 934	32	12A/12B	14A/23B/21C
Days	15	Around 200	14A/21B	22A/12B/17C
Activities	16	Around 10	10	8
Algorithms	Naive Bayes	AR + AD	HMM + ANN	HMM, CRF
Types	Multiple	Multiple	Binary	Binary

We prefer the method of the Ordonez ADLs dataset donated by the Machine Learning Repository of UCI (University of California, Irvine, CA, USA) [13,26] on 28 October 2013, because the number of sensors and activities might be better suited for deployment in a real home environment. The Ordonez dataset includes leaving, toileting, showering, sleeping, breakfast, lunch, dinner, snack, spare_time/TV, and grooming. The sensors of Ordonez are of pressure/PIR type, which could be more accurate to identify the resident’s pattern. The way Ordonez deploys the sensors seems more practical for an actual smart home, because the common behavior is not much within a home level setting. Thus, making full use of a wireless network and installing the sensors indoors and specific wares is enough to learn the associations between activities and sensors, rather than infer the user’s next action with conditional transition probability.

### 3.2. Time Sensitive Parameter

The main idea is to process the dataset at lower computing cost and with nearly real-time response, using a definition drawn from Viktor Mayer-Schönberger: that is, simple algorithms running on a big dataset perform better than complex algorithms run on a small dataset [37]. Therefore we try to map the raw dataset into a sparse matrix, with a time-slice intervals of 1 minute, in order to enhance the dimensions and reduce the complexity for a cost-effective embedded application.

Some social science researchers have found that human behaviors might be strongly time-sensitive, and there might be a routine daily or weekly pattern, as is shown in Kasteren activities [12]. Thus, we use the hypothesis that there is a correlation coefficient between human patterns and time. To verify the idea, we tried to explore the activity routine in the Ordonez dataset.

**Figure 1:**
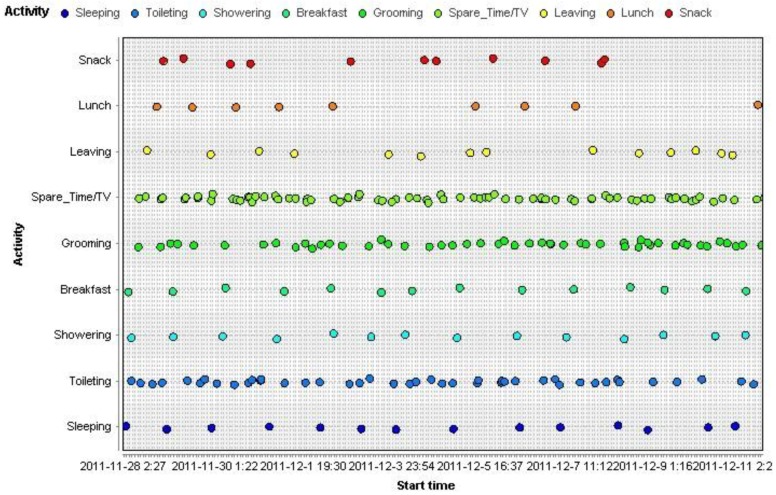
Frequency of performed activities in Ordzonez dataset for one week.

We use RapidMinner 5.3 to plot the activity corresponding to start time within the dataset, as shown in Figure 1, with the frequency performance as the intensity of scattered points. Obviously, most of the behavior fits a regular pattern. We believe that most behavior is time sensitive so the time axes play an important role in activity recognition.

We chose the fuzzy set function to map the time value into float numerical type; let *T* be the time parameter, and X be the real value of timestamp, so we extract the time value as *T:X → [0, 1]*. Other parameters like weekday, month, year and so on, are stored as a specific column, which is discussed in Section 3.3 and Section 3.4 in detail.

### 3.3. Superset Transformation

In order to match the sensor data and time value better, we used the Superset Transform [38] to set the original dataset lossless mapping to the new sparse matrix with a uniform time axis. Meanwhile, we checked the start_time and end_time of the original dataset of each sensor and activity, and match them with the time axis. Thus, the actual deployment period of the private cyber physical system would be more flexible, as each sensor accesses only a column. Once the signals are sampled by the sensors, information is sent to the main control node and the node as a server works to listen and record the time and signal together.

**Figure 2:**
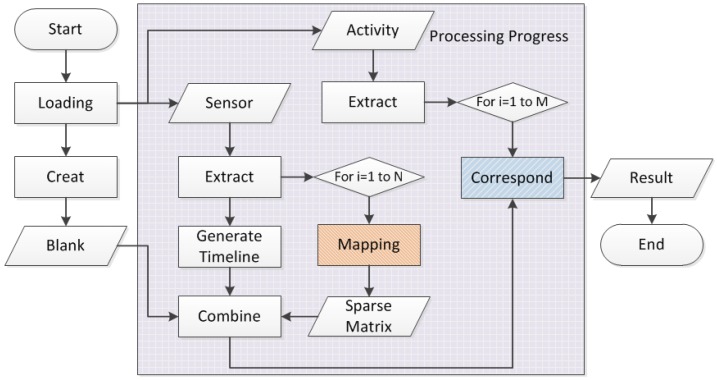
Abstract flowchart of the mapping function.

The whole transformation process is elaborated in Figure 2. Firstly, it scans the sensor dataset to get the row number w, and extracts the earliest Start time and the latest End time to generate the timeline, in 1 min steps. Then the loop process traverses the mapping module to find each unique sensor id as a new column name record in the sparse matrix, with an initiated value of 0 for each. At the same time in each loop cycle, the function extracts the Start time and End time for comparison, and we change that format into a standard float value so that the process would be easier. In addition, the sensor value is set to 1 if the corresponding sensor is in active status between the Start time and End time, thus, the sparse matrix is generated according to the time line. The secondary loop process is to deal with the activity dataset. Extract the row number, and then to loop each row to adjust the Start time and End time and record the activity together correspond to single time line and additional column attach the activities label tag. Finally, it removes the integer part off the float time value to retain only the decimal part, thus completing the transformation of the fuzzy set and the mapping process, as Figure 2 shows.

Thus, we mapped the raw dataset into a new sparse binary sensor matrix, with an additional timeline with float value. We tried to explore whether weekdays affect the behavior pattern, and we got one step further mapping the dataset; the results and analysis are shown in segment 4 on the left column and activities labels on the right column. Also, the dimensions depend on both the number and status types of the sensors.

### 3.4. Classifier Selection

After Super Set Transformation, we tried the new data form of both the KasterenA and OrdonezB dataset (longer sampled actual living data) through a 3-fold cross validation to verify the theoretical activities recognition accuracy of our prototype. Details of the results are described in Section 5.6.

Considering the performance and complexity, and to prevent over fitting, we tried to get a proper model that is able to be deployed on Raspberry Pi (ARM7 with 512 MB memory) to serve as the main control and computing node of a smart home. We use the time consumption to describe the complexity, considering the performance of Raspberry Pi, real-time feedback, and overall accuracy; we selected the decision tree algorithm because Naive Bayes has a higher precision error rate, while the artificial neural network and k-NN are too complex for pervasive devices, although they have finer features. The parameters comparison is described in Table 2.

**Table 2:** Comparison of classifiers run on Raspberry Pi.

Algorithm	Naive Bayes	Decision Tree	k-NN	ANN
Accuracy	97.38%	98.52%	98.05%	98.58%
Time Complexity	1 s	12 s	1 min, 47 s	6 min, 32 s

### 3.5. Sensors Selection

Sampling human behaviors from sensors, environmental data and activities collection are required. For surveillance movement of the householders, passive infra-red (PIR) sensors with 10 μm wavelength satisfied the demand. To collect the environmental data, we needed sensors to gather temperature, humidity, light, gas, smoke, and so on. In order to obtain the actual operation to control the electric appliances of the living human, touch sensor and mobile control records are necessary. Taking comprehensive consideration of robustness, power consumption and practical deployment of wiring and decorate works, we chose the sensors below and transmitted the data through a TI-cc2530 based ZigBee development board.

#### 3.5.1. DHT11

**Figure 3:**
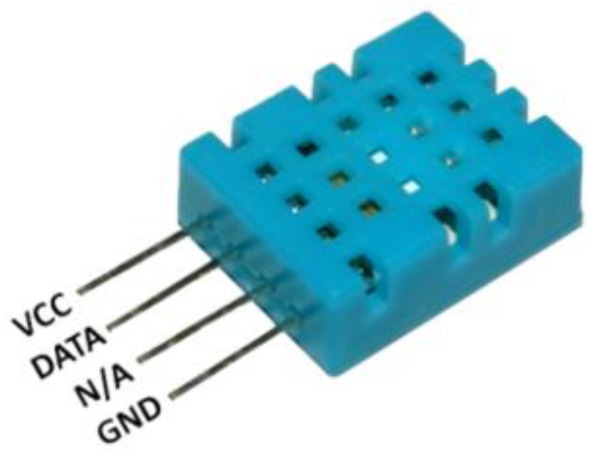
Temperature and humidity sensor module DHT11.

DHT11 is a temperature and humidity sensor module, as shown in Figure 3. For temperature, the measurement range is 0~50 °C, and precision is ±1 °C. For humidity, the measurement range is 20%–90% RH, plus precision ±5% RH. This is simple and stable for home level utility.

#### 3.5.2. MQ-2

**Figure 4:**
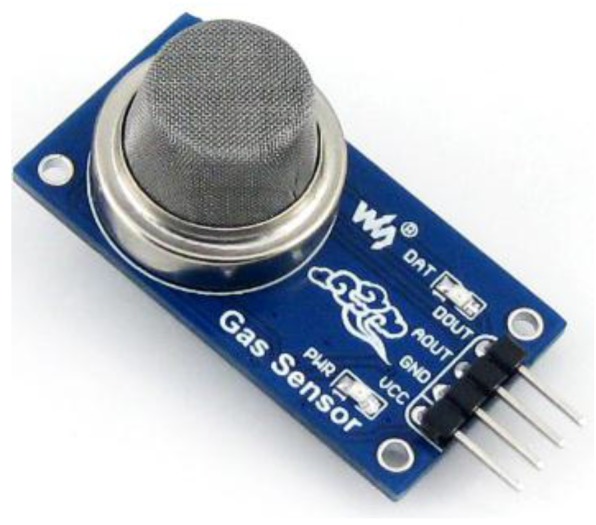
Smoke sensor MQ-2 module physical map.

MQ-2 is a gas sensor module capable of detecting methane, propane, hydrogen, alcohol and liquefied petroleum gas, as shown in Figure 4. The detection range is 100~20,000 ppm, and an operating temperature range of −10 °C–50 °C with relative humidity of 65% ± 5% RH, which is simple and stable for home level utility.

#### 3.5.3. BH1750FVI

**Figure 5:**
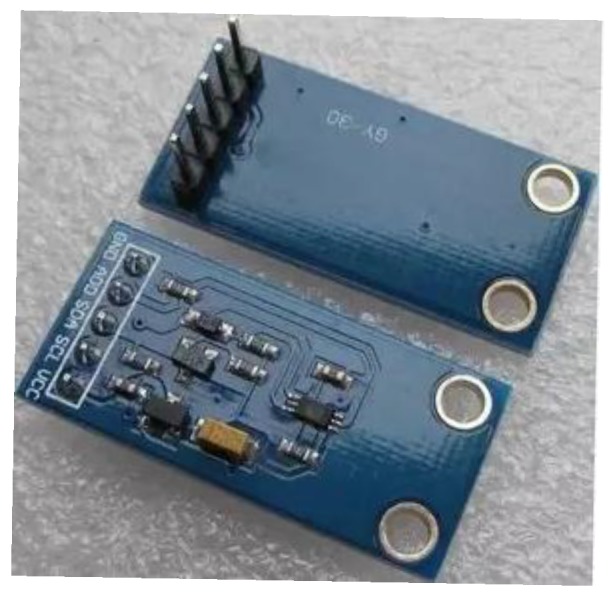
Light sensor BH1750FVI module physical map.

BH1750FVI, shown if Figure 5, is a digital ambient light sensor IC module. It is possible to detect a wide range at high resolution (1~65535 lx). The operating temperature range is from −40 °C to 50 °C, which is simple and stable for home level utility.

#### 3.5.4. HC-SR501

**Figure 6:**
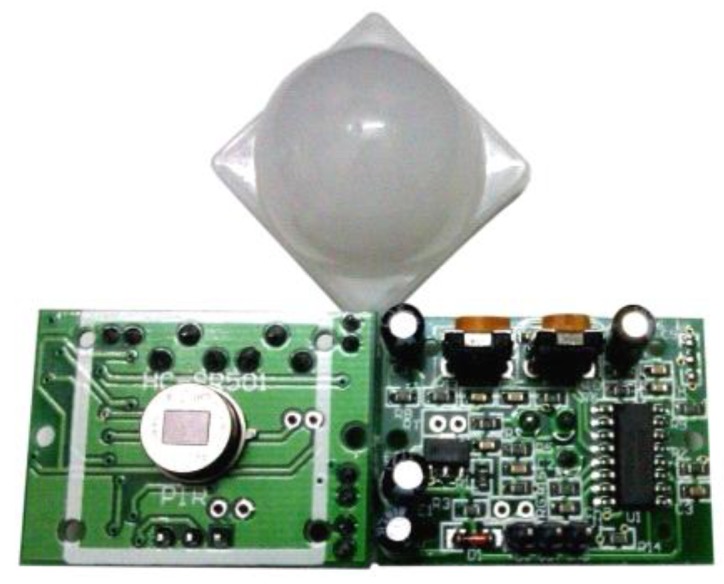
HC-SR501 human infrared sensor module physical map.

HC-SR501 is a human body sensing module, as shown in Figure 6, using 10 μm wavelength infra-red to detect human body movement. The operating temperature range is from −15 °C to 70 °C, and the effective distance less than 15 m, sufficient for in one room in a smart home.

#### 3.5.5. TTP223

**Figure 7:**
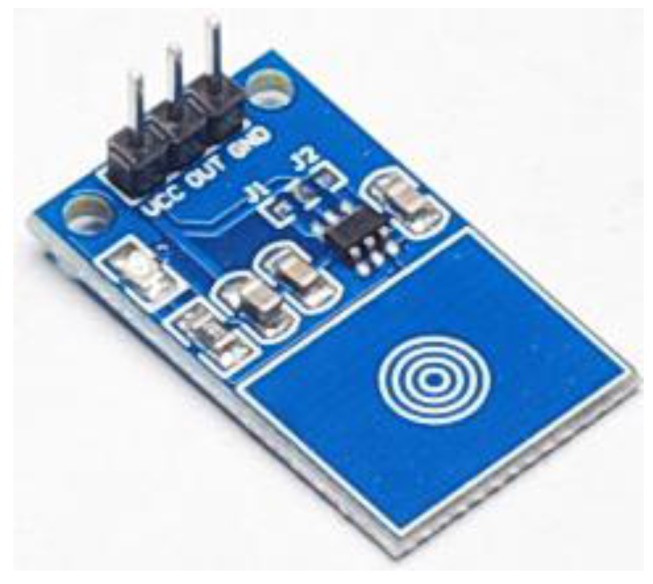
TTP223 human touch module.

TTP223 is a human touch sensor—a capacitive touch switch, as shown in Figure 7. We used TTP223 to replace parts of the original light switch on the wall. The operating temperature range is from −20 °C to 70 °C, and the effective response time range is from 60 micro second to 4 s for readjustment, capable for normal human living behaviors.

#### 3.5.6. DSM501A

**Figure 8:**
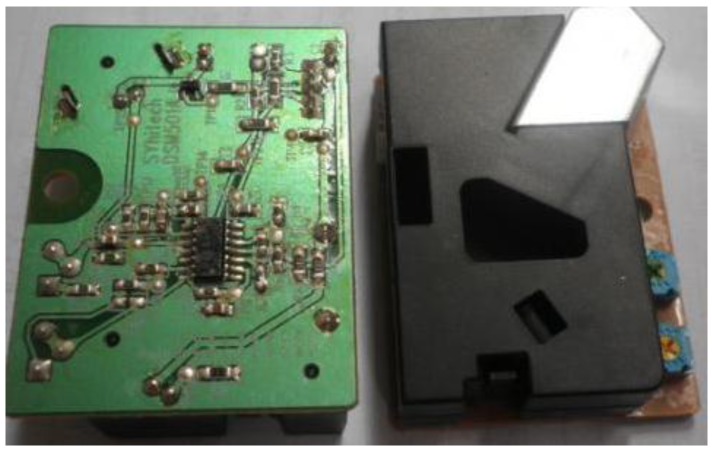
DSM501A particles detection module.

DSM501A, shown in Figure 8, is an optional dust sensor that can detect particles of 1 μm in diameter, which guarantees the living air condition for the residents. We use DSM501A to detect the value of PM2.5 and to determine the ventilation. The operating temperature range is −20 °C~80 °C.

## 4. Prototype Design

With the rapid development of electronic products and the popularization of the network, smart home appliances, IP cameras, smart plugs, electric relay has enabled many auxiliary control functions. However, in most cases, residents have already decorated their rooms and are not willing to change their original layout to do the wiring work. Thus, we propose a heterogeneous wireless architecture that considers both cost-effectiveness and assist-control functions, respectively. First of all, sensors integrated with cc2350 as a front-end device to sample the living data in each room can be easily attached to the wall, and work via a battery. Secondly, the back-end server sends commands to control the appliances via Wi-Fi. In this way, residents only need to configure and initialize it after the installation of our sensing home solution.

### 4.1. Architecture

In our scheme, a cc2530 master node is required in the living room, the master node integrates an ESP8266 IC, which enables the serial ZigBee signal received to be transmitted in Wi-Fi protocol. The living room is typically located at the center of each house, so that every cc2530-based slave sample front-end device equipped with sensors is able to transmit their data through ZigBee within a reasonable distance. Then, other rooms like the bedroom, garage, kitchen, and study room where each requires one front-end device to gather the living information and environmental parameters.

**Figure 9:**
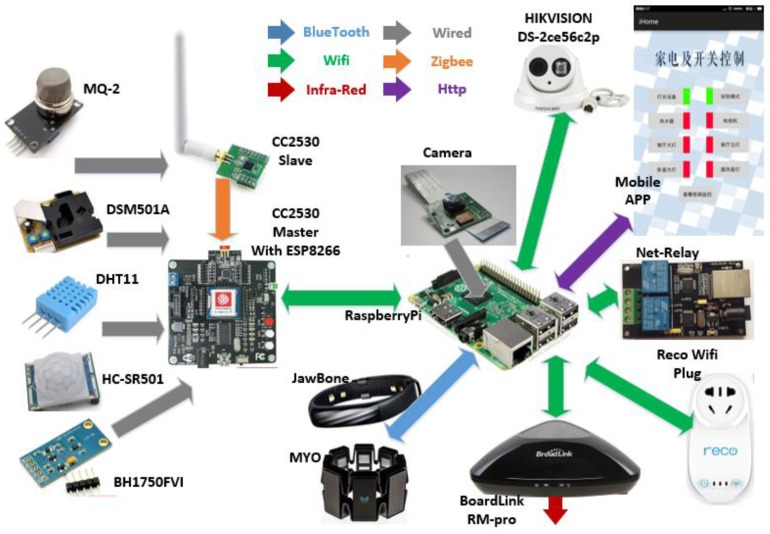
Draft architecture of the sensing home.

At first, we used one X86-based PC workstation as our server to collect, store and process the data we sampled, and we tried to control our appliances via a smart Wi-Fi plug or relay. As the number of the plugs increased, we found that every smart device has its control code and APP, and each brand of device is isolated but extensible. Moreover, the PC server only needs to listen to the sampled data, process the data with a trained classifier, and return the feedback control message to the destination; the occupation rates of CPU and memory are low, which means a waste of electric power. Finally, we decided to evaluate the workload and scalability peripheral equipment of a modern smart home, and tried to figure out a feasible wireless scheme, as shown in Figure 9. For remote control and surveillance, a mobile app with a cloud server to map the home IP address is required. As illustrated with the purple arrow, the mobile app uses json format communication via http protocol. In the home, Wi-Fi is capable for video or other short distance communication devices, as shown with green arrows. We used Raspberry Pi as the main control node server, because it is sufficient to deploy Linux OS to run our programs and costs much less than a PC workstation. In addition, optional wearable devices are easy to be incorporated into our sensing home if Bluetooth interface of the USB slot is extended, as the blue arrow shows in Figure 9. We tried four awesome wearable devices: Jawbone, Apple Watch, MYO and Microsoft band because they open their data interface or API for developers. With these equipment, we are able to collect the movement, step, heart rate or gestures of the residents for finer features.

### 4.2. Heterogeneous Wireless Network

ZigBee is a kind of low-speed, short-distance transmission protocol based on the IEEE 802.15 standard. Related to environmental safety, the features of ZigBee perform well enough for home care service applications. Firstly, the transmission speed is 10~250 Kbps, and also, the distance range is about several dozen meters, capable for sensors in intelligent home settings except for a vision-based scheme. Characteristics of ZigBee were illustrated by Yi *et al.* [39]: saving energy, high reliability and high expandability. Thus we designed our front-end sensing box to sample the living data and used a cc2530-based ZigBee scheme to transmit the Byte-level sensor data to a home server.

Wi-Fi is also a wireless local area network based on the IEEE 802.11 standard, with 100 m transmission radius and maximum 54 Mbps speed which enables video, mobile, electrical control and communication, public wireless Internet access and many wireless product usage. Lee *et al*. [40] described the features of Wi-Fi: standard maturity, high recognition by public, low interference. With these features, we used the Wi-Fi protocol to control the smart devices, but transmission of the video from an IP camera is also possible. We chose Raspberry Pi as the main home server, with a RTL8188CUS 802.11N WLAN adapter through a USB interface as AP mode to establish our own WLAN.

The Bluetooth technology, with features lower power consumption, shorter distance wireless transmission and ease of use, is widely adopted by wearable devices with mobile applications. We used the BCM2045 Bluetooth USB adapter and system supported drivers: apt-get install bluetooth bluez-utils bluez-compat blueman.

In the custom-made front-end sensing box, we adopted a ESP8266 to transform the ZigBee format to Wi-Fi, and at the back-end server, we used USB interfaces plus WLAN and a Bluetooth adapter. Thus, the Heterogeneous Wireless Network of a sensing home was established.

### 4.3. System-Level Design

**Figure 10:**
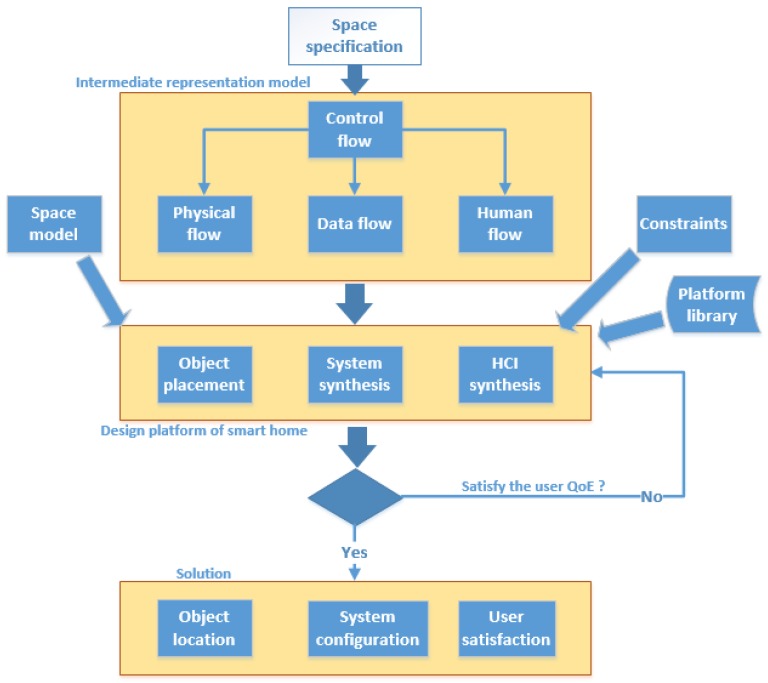
The design framework for sensing home.

System-level design refers to the refinement from application specification to prototype implementation, according to given constraints. As shown in Figure 10, we present a flow-based model, which allows the formal capture of the relations between human actors and cyber-physical objects. It consists of control flow, physical flow, data flow and human flow to guarantee the quality of experience (QoE) of the residents. We used a system-level design method to refine our work as described in Section 5.

Initially, the space specification is defined according to the requirements of the application. Subsequently, the intermediate representation model divided the specification by the control flow, corresponds with data flow, physical flow and human flow. Furthermore, the specification is mapped into the design platform of our sensing home, leveraging the intermediate representation model, considering the constraints and space model. Finally, this solution process is iterated until the QoE is satisfied.

## 5. Implementation

**Figure 11:**
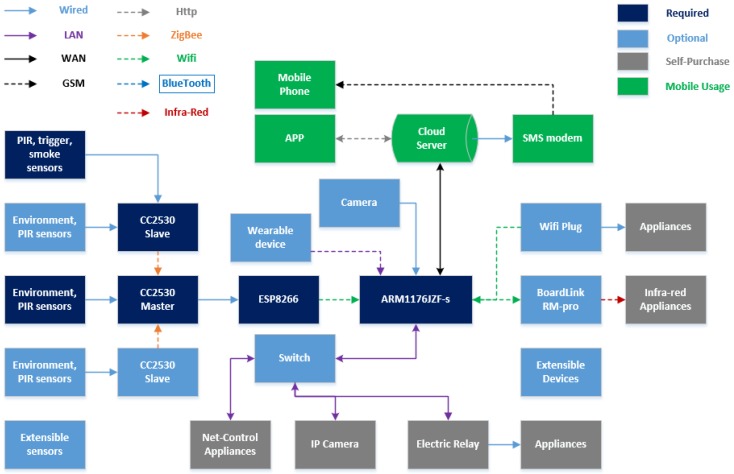
Architecture and module connections of sensing home.

In order to demonstrate the feasibility and effectiveness of the prototype we proposed, the deployment of a real home environment is required. We purchased the sensors mentioned in Section 3, wired with a TI-cc2530-based development board, and both slave node and the master node are able to sample the living data. An ARM11 based Raspberry Pi served as the main control node, which took charge of storage, computation workload, WLAN AP, router function and feedback control. In addition, it was connected to a public cloud server. As shown in Figure 11, the minimum equipment of a sensing home required is marked as dark blue, including PIRs, triggers, harmful detection, environmental sensors integrated with a front-end box, and an ARM-based server. For extension with more front-end sensing boxes in a real house, smart devices, an IP camera, infra-red appliances, Wi-Fi plug and relay are optional, as shown in light blue blocks. The home appliances shown in grey blocks, which belong to the resident, such as TV, air conditioner, electric kettle, bulb and exhaust fan, must be wired with Wi-Fi plugs or relays for auto control. In addition, a mobile app enables the user to check the environmental information and status of electrical appliances, remote control and alarm when necessary. Detailed module linkage and communication protocols are shown in Figure 11.

### 5.1. ZigBee Front-End

Sensing box is the sampling front-end device, to collect the living and environmental data from sensors. The slave nodes, required for each room, can be attached to the wall and work with a battery. For the PIR sensors, the frequency of sampling is 0.2 Hz. For the environmental sensors, a time interval of 1~5 min is enough for utilities and saves as much energy as possible. Data is transmitted through the TI-cc2530-based IC via ZigBee Z-stack to the master node.

ZigBee protocol is divided into five layers: the physical layer (PHY), media access control layer (MAC), the network layer (NWK), application support sub-layer (APS), the application layer (APL). ZigBee protocol is divided into two parts; IEEE802.15.4 defines the physical layer and MAC technical specifications, and the ZigBee Alliance defines the network layer, security layer and application layer specification, a function of realization, and provides the user with a number of application layers, API.

The application support sub-layer is to provide a number of API functions, in addition to the binding table, which is also stored in the application support sub-layer. The ZigBee device object is the running port 0 ZDO applications, mainly to provide some network management functions.

**Figure 12:**
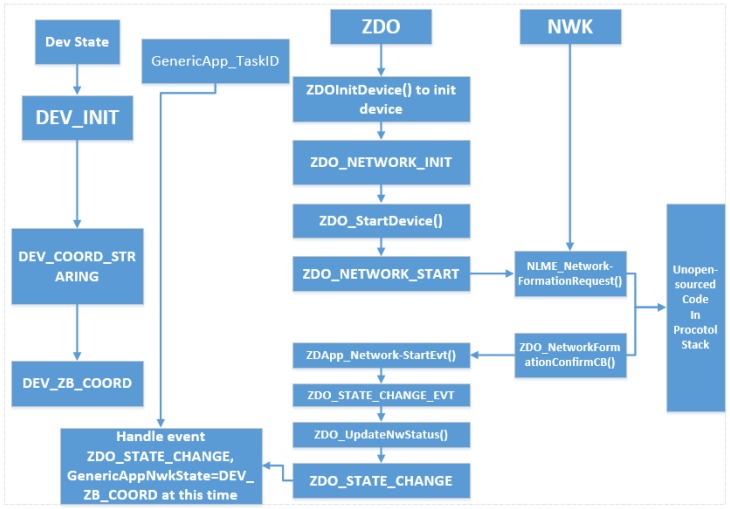
Coordinator networking process.

As can be seen from Figure 12, the process of establishing the network is ZDO to achieve, after the network is established, the application layer will receive ZDO_STATE_CHANGE messages, and the message contains the current network status of nodes, using GenericApp_NwkState = (devStates_t) (MSGpkt-> hdr.status); it can read the current network status, as the coordinator, the network status is DEV_ZB_COOR after the network setup.

The master node should be deployed at the center of the house, mainly the living room because the actual radius with wall attenuation is about 25 m. In addition, the master node work with power adapter because it transform the data from ZigBee to Wi-Fi signal via the ESP8266, which means more power consumption no longer fit a battery scheme. The master node gathers all the sensing data and transmit it to the main control server.

### 5.2. Activity Recognition

**Figure 13:**
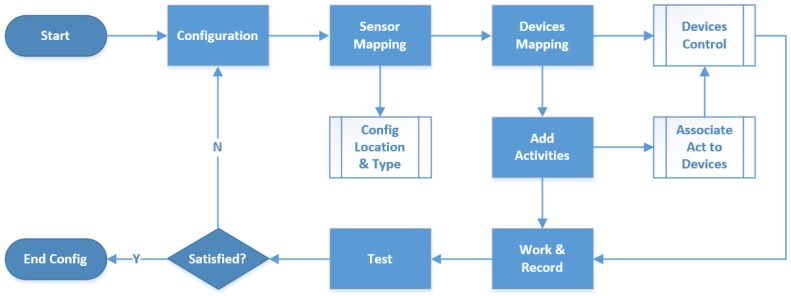
Flow chart of configuration module.

After the installation of sensors and the front-end box, the first step of the main server is to configure the sensors and the appliances. In order to be compatible with more sensors and devices for our scalability, two mapping libraries are required. One library is for the sensors, which defines the type, version, function, data format, and parameters. Another library is for the devices to control, such as Wi-Fi plugs, relays, TVs, and air conditioners. The configuration module maps those settings, matches sensors and devices by ID, adding raw routine activities of the resident on, associating the activity with the corresponding control logic. All the sensors and devices are naturally clustered by room to be associated with human activity, and an iterate test program is necessary to achieve better QoE. A flow chart of the whole configuration module can be seen in Figure 13.

Following common practice, the actual living data collection of the resident is stored as the training set to recognize the activities and the routine habits. The more the residents use our sensing home system, the higher the accuracy of the behavior classifier. Both adjustment and modification are supported to refine the application through a browser operation connected straight to the home server via WLAN.

### 5.3. Feed-Back Control of Appliances

**Figure 14:**
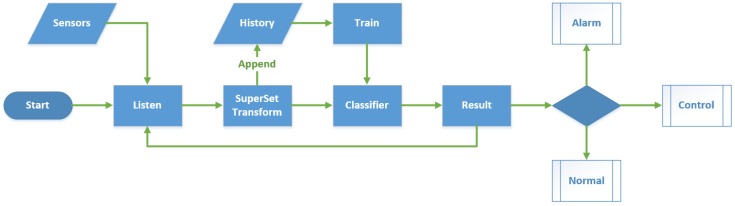
Raw control logic of sensing home.

**Figure 15:**
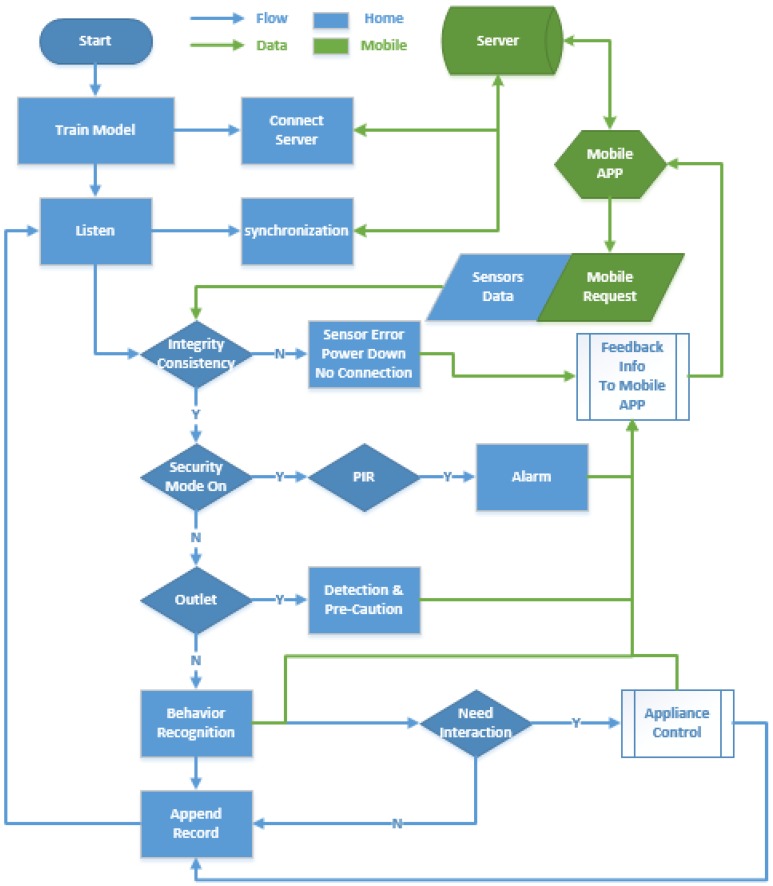
Detailed feedback control flowchart of sensing home.

The main program running on Raspberry Pi is to listen to and respond to the data from sensors. As shown in Figure 14 and Figure 15, the home server starts and initializes the configuration, and connects its IP address to a cloud server. Then, it waits for the data transmitted from the sensors and returns the reasonable feedback following the original configuration. There are four classes of functions that we provided: (1) Self-checking, verifying the integrity and consistency of the sensors and devices status in order to inform the user whether any device not working, at each restart and 6 h interval; (2) An alarm, to detect gas leaks or fires. Moreover, when a user leaves home and the security mode is on, if there is any break-in or an intruder, it will activate the PIR sensors, inform the resident and the police; (3) Activity recognition: using the trained classifier to determine the auxiliary control of electrical apparatus, like turning on lights or an air conditioner; (4) Outlet detection: we used the DBSCAN algorithm and statistical normal distribution to extract abnormal behavior and send a precautionary message to appropriate people. Cardio-cerebral vascular disease, stroke and falls lead to the outlet behavior, for a rescue from these conditions, and for the greater probability of survival.

### 5.4. Synchronization with the Cloud Server

In order to achieve remote control and observation of home appliances, a cloud server and database are required for the IP addresses and status mapping. We have adopted a relatively high profile Ali cloud server, which is similar to the Amazon Web Services (AWS) public cloud services in China. An Ali cloud server-based independent research and development of large-scale distributed computing systems through virtualization technologies across IT resources, features possible self-management, data security and other advances.

The software environment of the cloud server we used is 1 Mbps bandwidth, CPU 1 core, 4 GB memory, Ubuntu 64 bit operating system, equipped with apache php5.2 and Mysql5.3 database. It provides a basic environment for elastic developing extensions.

Based on the software and development environment, our cloud service and smart home architecture has been established. We use PHP scripts to write the server program, and deploy the program on a cloud server, for data processing, analysis, transmission and storage. Basic features of sensing a home cloud server include:

(1) Receiving a command from an app, analyzing the command, storing it in the database and sending it to the user’s home Raspberry, for the home control.

(2) Sending the information from a database to the app to show to the householder, so that users can be observed in real-time in the home situation. On the cloud service side, the information about appliances and sensors are stored in the cloud database through php program that will send it to the app side, to achieve a real-time display effect.

(3) Receiving status data from home sensors and electrical appliances. Because changes in home appliances and sensor data occur in real time, there is intermittent access to information, and distribution of the data from the home Raspberry Pi, stored for synchronization.

**Figure 16:**
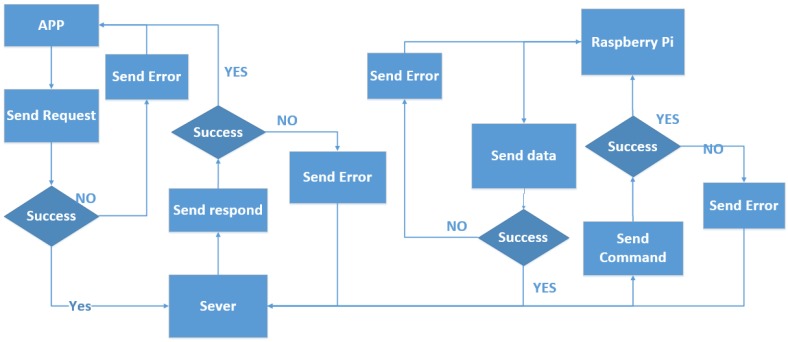
Synchronization flowchart.

When the server communicates with the app, we use JavaScript Object Notation (JSON) format. The flow chart of synchronization from the home server to the mobile app via cloud server is shown in Figure 16.

With the increase in the volume of users, the architecture of a load-balanced cluster to provide services would be required in the future for expanding the size of the cluster. A server-based management model of the original stand-alone can no longer meet the demands, so new requirements must be centralized, with packet volume, automated management, and capable of mass execution scheduled tasks.

The core LVS (memory cache pool) is the scheduling node, and the node is responsible for the scheduling algorithm dispersed to flow through each node. Due to scheduling the consumption of scarce resources, it is possible to produce a high throughput rate, with greater number of nodes.

**Figure 17:**
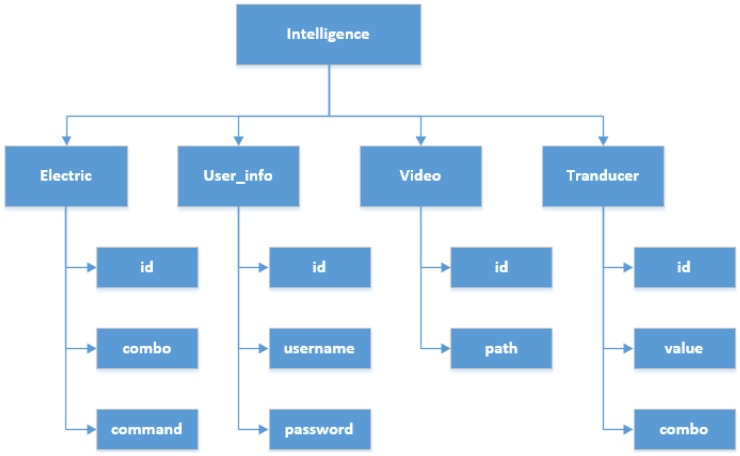
Detailed segments and architecture of the cloud database.

Overall, the cloud server acts as a bridge in the smart home, and the connection from the exchange of information at home and the app, allowing users to know the information from the home. The feature list is shown in Table 3 and the database architecture is shown in Figure 17.

**Table 3:** Features of cloud server program and mobile synchronization.

	Recipient	Sender	Method	URL	Data Name	Data Format
Check status	App	Cloud server	POST	../ele_show.php	Id;command;combo	json
Change status	Cloud server; Raspberry Pi	App	POST	../ele_update.php	Command;id	Json;string
Add sensor data	Cloud server	Raspberry Pi	POST	../tran_add.php	Value;id;combo	string
Check sensor data	App	Cloud server	POST	../tran_show.php	Value;id;	json
View pictures	App	Cloud server	POST	../video_show.php	Id;path	json
User login	Cloud server	App	POST	../user_login.php	username,password	json

### 5.5. Mobile APP

There are several platforms for developing smart phone applications such as Windows Mobile, Symbian, iOS and Android. In the proposed system, most of the phones and hand-held devices support Android OS. In spite of the developing environment, we can only analyze the modules of different functions from the service logic. The software configuration is illustrated as follows:

Operating system: Windows 8.1:
Android API: Ice Cream Sandwich
Serial number: API 14
Version: >= Android 4.0:

The designed app for the smart home system provides the following functionalities to the user: (1) Remote connection (via internet) to the smart home micro web-server on Raspberry Pi (with software configuration of Linux, Nginx, SqLite and PHP as a home server); requires server real IP and user authentication; (2) Device control and monitoring; (3) Scheduling tasks and setting automatic control of the smart home environment; (4) Password change option; (5) Supports voice activation for switching functions.

**Figure 18:**
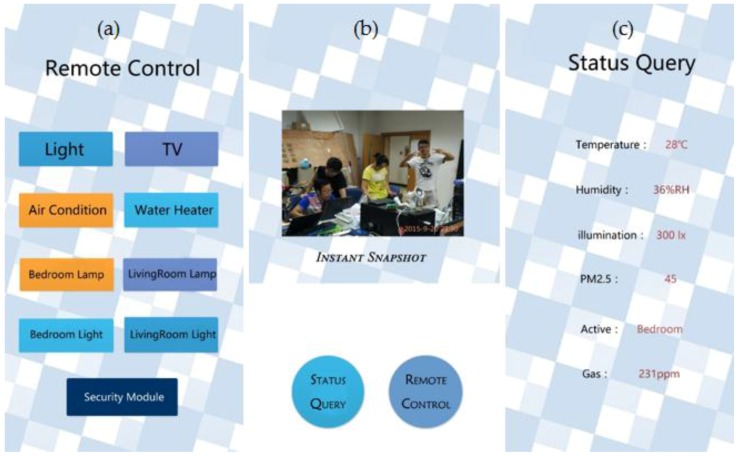
(**a**) Remote control GUI; (**b**) Snapshot camera of front page; (**c**) Status query GUI.

**Figure 19:**
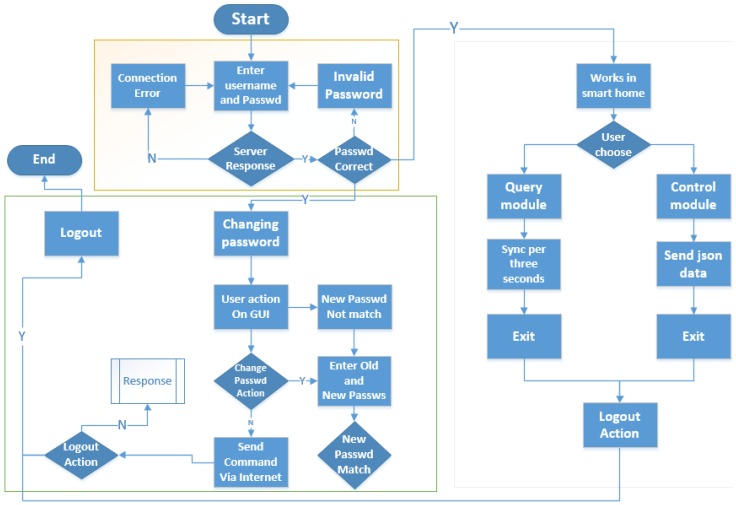
General processing of proposed smart home app.

For Android, Java programming language using the Android Software Development Kit (SDK) has been used for the development and implementation of the smart home app. The SDK includes a complete set of development tools such as debugger, libraries, a handset emulator with documentation, sample code and tutorials. Android Studio (running on the Windows 7 and 8 development platform), which is the officially supported integrated development environment (IDE) that has been used in conjunction with Android when Google officially confirmed that their Android group had canceled the support of Eclipse in Android developing. Development Tools (ADT) Plug-in is used to develop the smart home app. The screenshots of the smart home app developed are shown in Figure 18, while the processing of the smart home app is shown in Figure 19.

When it is necessary to connect and access the smart home micro web-server, the user does not have to enter the real IP address of web-server. What the user needs to do is to enter a username and password, which is set and configured by after-sales technical support. In the configuration, technical support is supposed to match the distinct server IP with the cloud server IP and their username and password.

When the micro web-server grants access to the smart home app, the response packet containing the response code will be received, which means the status of the connection or information of the error during connection. The app processes the response packet to determine the micro web-server’s response. Response code 200 indicates that the password is correct, and the app will switch to the main page, which is the navigation to another page, including a query page and control page.

In the query page, the app automatically synchronizes and updates every three seconds by a default time interval, using the data from the response packet to reflect the real time status of the smart home devices (Table 3). The frequency of query can also be set by users. The response code and devices with their status are packaged in the format of JSON, whose format is “key: value”, similar to xml, which is now in extensive use. The general response packet structure is shown in Table 4. A two-level format of JSON is needed because there is more than one device in the whole system. For the next level of JSON, it contains the information of a different device.

**Table 4:** Structure of mobile server packet.

Level	Key	Value	Type	Definition	Comments
1	c_id	9527	String	Id of Master node	
1	status	200	int	Connection status	200: ok, 404: wrong password
2	device	light	String	Device name	
2	Id	01	int	Device Id	
2	state	1	int	Device status	1: on, 0: off

In the query page, when the app automatically synchronizes and detects that there is warning information in the response packet, the smart home app is designed to push a warning notification on the notification board. In this way, users are able to tap the notification to see the capture of the camera, which is usually a picture. Thus, home owners are notified and are able to handle break-ins, fire detection or life-threatening problems. The smart home system was fully functional for switching applications, and as the appliances are switched on, the user interface is updated to reflect the current status. The smart home system was also tested for intrusion and fire detection, whereby it successfully detected the respective events, generating an e-mail to the user and turning on a siren. A notification would be received by the user on the mobile device.

On the control page, the user can perform the desired action from the GUI when access is granted. The password can also be managed by the user from the smart home app. If the new passwords match then the command packet containing the new password is sent to the micro web-server. If the password is successfully changed, the response code 201 will be received.

The communication with the web server in the control page is similar to that in the query page. The general response packet structure and the status codes represent a different status of the last control task, which is specifically shown below in Table 5. However, what is different is that only one level of JSON is built in this structure because the user only controls one device at a time. The device and id identify every device, and state represents the status of the device that the users want to control in the system. Zero indicates off state while a one indicates on state for the status for switching functions.

**Table 5:** General response packet structure and codes of status.

Level	Key	Value	Type	Definition	Comments
1	c_id	9527	String	Id of master node	
1	status	200	int	Connection status	200: ok, 404: wrong password
1	device	light	String	Device name	
1	Id	01	int	Device Id	
1	state	1	int	Device status	1: on, 0: off

In the control page, the security mode can also be activated where the smart home environment will be controlled the same as when there are no people in the house—for example automatically turning certain lights on/off during the night/day, and sending a warning to the public security and the home owner.

The proposed system has all the features with respect to mobile use. On the other hand, it also has security features such as user authentication for accessing the smart home system, and intrusion and fire detection with alert notification.

### 5.6. Results

**Figure 20:**
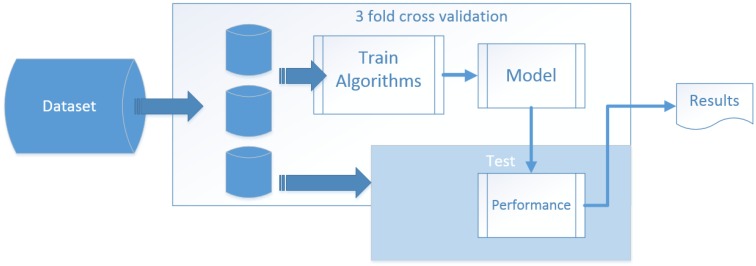
Processing flow chart of 3-fold cross validation.

Due to the similarity of the Kasteren dataset and Ordonez dataset, they were collected, following the same ADL format; and reference [12] has verified that the inner parts of each dataset are basically the same, so we selected KasterenA and OrdonezB as experimental data because they have a longer series of observed samples. Thus, we are able to use the same processing flow with different intelligent algorithms to compare the observation results. The whole process is shown in Figure 20. We tested 3- to 10-fold cross validation, and the result is nearly the same, a set x value of 3 is enough for the experiments.

#### 5.6.1. Accuracy

To further study whether a weekday parameter plays an important role, we mapped the dataset into the format, using the same processing flow; and the resulting accuracy for each algorithm is shown in Table 6.

**Table 6:** Results of accuracy, with or without weekday parameter.

Algorithm		Accuracy			
Data	Weekday	Tree	Neural Net	Naive Bayes	k-NN
Kasteren dataset	With	81.81%	90.11%	17.14%	90.26%
	Without	86.94%	88.6%	19%	87.5%
Ordonez dataset	With	98.76%	98.82%	97.23%	98.98%
	Without	98.24%	98.36%	98.01%	98.33%

Overall, the Ordonez scheme performs better than the Kasteren one, but that is due to the difference between deployment methods. Unfortunately, neither the Kasteren nor Ordonez dataset showed proof that a weekday parameter is important, but we still believe strongly that households are likely to have an association between certain activity and a weekday in actual deployment, like watching a football game or other social activities, so in other experiments below, we discuss the dataset adopted with a weekday parameter.

#### 5.6.2. Cost Comparison

Power consumption (average):

Raspberry: 1.67 W/h
Master node: 0.82 W/h
Slave node: 0.005 W/h

The total power consumption is about 2.5 watts per hour (without a video camera), and the total cost of hardware devices is about $120, which is affordable for consumer electronics. As vision- based activity recognition solutions involve a huge computational task, deployment is likely to require many video cameras with PC workstations or stronger embedded devices. For fair comparison, we list each kind of single product to compare the total cost of each, as shown in Table 7. Using binary sensors with our heterogeneous wireless scheme is able to save in the redecoration work, and is very cost-effective.

**Table 7:** Total Own Cost Comparison.

Device	Power Consumption (W/h)	Price of Hardware ($)
Raspberry Pi	0.5~3.5	25–30
Our master node	0.82	15
Our slave node	0.005	10
Video camera	10~12	>20
Laptop	40~70	>200
PC workstation	>150	>200
Embedded video box	>12	>30

### 5.7. Privacy and Interference

In the phrase of testing and verification on our prototype, we focus on the accuracy, performance, power consumption, and more importantly, user experience. There are potential threats concerning private data protection and 2.4 GHz wireless interference. To solve these problems, we put forward a feasible protection and coexistence mechanism according to hardware limitation.

#### 5.7.1. Privacy Protection

We use three ways to protect the security of data. First, all the data of the residents is stored in their home. Our cloud service only provides the bridge to set up the point to point access from mobile phones to the Raspberry Pi at home. No extra data storage resides on the cloud except IP, port, usernames and passwords. Second, an encryption mechanism is used on both software and hardware. For the software level, we use an AES algorithm to encrypt the information, where the secret key is relevant to the unique serial number of the Raspberry Pi, user password and PIN code. For the hardware, taking the computing capability into account, we use cyclic shifting to further protect the data and the key is related to the serial number of our product. Third, we provide an optional PIN code lock on the APP, and 3–5 times failed access attempts lead to the account being frozen in order to prevent other people from using the smartphone of the administrator.

#### 5.7.2. Wireless Harmony

Generally, both Wi-Fi and the ZigBee protocol work at 2.4 GHz, so using the default setting leads to conflict and interference. Setting the Wi-Fi AP to work at 5 GHz while leaving ZigBee at 2.4 GHz is one possible solution. Furthermore, we have designed a coexistence mechanism at the software level, setting the priority of ZigBee higher than the WLAN because TCP/IP has a retransmission mechanism. When a conflict occurs, the data of the sensors at byte level with 0.2 Hz transmission by ZigBee is guaranteed preference, so the experience of the residents will not be affected. In extreme cases like over 14 Wi-Fi AP in the same room, or under electromagnetic interference shielding conditions, our devices might not work, but these situations would hardly happen in a realistic home setting, and there is wireless signal attenuation due to the walls.

## 6. Conclusions and Future Works

In this paper, we have compared open smart home projects, and researched the differences between each scheme to find a practical, intelligent home furnishing scheme and mobile utility. This paper introduces a new idea for a sensor and automation system to implement for activity recognition in a house setting: We rebuild the ADL as a sparse matrix to collect each parameter in different columns, with front end mapping for sensors and wearable devices and backend mapping for home appliance control codes. We propose a heterogeneous wireless solution to reduce the wiring and decoration work, with features of cost-effectiveness, energy-effectiveness and compatibility with more brands of appliances and sensors integrated with a mobile app. We experimented with probabilistic models that transform a public ADL dataset in order to reduce the process cost for embedded usage with high time-slice accuracy. However, a cold startup issue is still not resolved yet, and additionally, residents have to tag at least one week of activity labels to train the model to reach a satisfactory level of performance. The design of practical applications on an intelligent router is under way, and we may make our own dataset of experimental data, products and mobile apps public in the future.
